# Interaction between genistein and the endothelial activation and stress index in relation to estimated 10-year atherosclerotic cardiovascular disease risk classification

**DOI:** 10.1371/journal.pone.0356477

**Published:** 2026-08-19

**Authors:** Xinru Guo, Yijie Lin, Shiting Mi, Yuqi Yin

**Affiliations:** 1 Department of Cardiology, Hangzhou TCM Hospital Affiliated to Zhejiang Chinese Medical University, Hangzhou, Zhejiang, China; 2 Jiangxi University of Traditional Chinese Medicine, Nanchang, Jiangxi, China; 3 Department of Geriatrics, Zhejiang Provincial People's Hospital (Affiliated People's Hospital), Hangzhou Medical College, Hangzhou, Zhejiang, China; Sichuan University, CHINA

## Abstract

**Background:**

The Endothelial Activation and Stress Index (EASIX) has been proposed as a marker of endothelial stress and may be associated with atherosclerotic cardiovascular disease (ASCVD) risk. Whether urinary genistein modifies this association remains unclear.

**Methods:**

Multivariable logistic regression examined the association between EASIX and estimated 10-year ASCVD risk classification. Effect modification by urinary genistein (<54.105 vs. ≥ 54.105 μg/g) was assessed using interaction terms and stratified analyses. Restricted cubic splines (RCS) evaluated dose–response relationships.

**Results:**

In the fully adjusted model, higher EASIX was independently associated with increased ASCVD risk (OR per unit increase = 1.407; 95% CI: 1.208–1.640; *P* < 0.001). A significant multiplicative interaction between EASIX and genistein was observed (OR = 1.66; 95% CI: 1.17–2.35; *P* = 0.005). Stratified analysis showed that this adverse association was slightly attenuated in the higher genistein group (OR = 1.360; 95% CI: 1.066–1.736; *P* = 0.015) compared to the lower group (OR = 1.397; 95% CI: 1.202–1.624; *P* < 0.001). RCS analyses showed a non-linear S-shaped association in the lower-genistein group (*P*_nonlinear_ = 0.020), whereas a predominantly linear relationship was observed in the higher-genistein group (*P*_nonlinear_ = 0.108).

**Conclusions:**

Higher EASIX was associated with elevated estimated 10-year ASCVD risk classification, and this association differed across urinary genistein strata. These findings should be interpreted as hypothesis-generating.

## 1. Introduction

With the accelerated aging of the global population, atherosclerotic cardiovascular disease (ASCVD) is placing a substantial burden on public health worldwide [1]. Endothelial dysfunction is widely recognized as a key pathological mechanism underlying the development of ASCVD [2–3]. However, despite the significance of endothelial function assessment in clinical research, current diagnostic methods, such as flow-mediated vasodilation, still face challenges regarding accuracy, reproducibility, and the lack of reliable biomarkers to effectively reflect endothelial status [4–6]. Recently, the endothelial cell activation and stress index (EASIX) has emerged as a novel biomarker and has been applied in prognostic studies in fields such as cancer, infections, and liver diseases [7–9]. However, its application in ASCVD remains underexplored, particularly concerning its relationship with ASCVD risk.

Additionally, genistein, a soy isoflavone, has been implicated in the development of metabolic diseases, neurodegenerative disorders, and cancer, potentially due to its anti-inflammatory properties and effects on apoptosis [10–12]. Evidence suggests that soy isoflavones may help prevent cardiovascular diseases (CVDs) by counteracting endothelial dysfunction [13]. Experimental studies have shown that genistein can improve the uncoupling of endothelial-type nitric oxide synthase, prevent pro-inflammatory factor-induced vascular endothelial barrier dysfunction, and inhibit the interactions between leukocytes and endothelial cells, thereby modulating vascular inflammation [14–16]. However, no studies have evaluated the association of genistein with ASCVD risk in the setting of endothelial dysfunction.

Therefore, this study aimed to examine the association between EASIX and estimated 10-year ASCVD risk classification and to assess the potential interaction between genistein and EASIX with respect to this relationship.

## 2. Materials and methods

### 2.1 Study population

In this cross-sectional study, data of participants were extracted from the National Health and Nutrition Examination Survey (NHANES) database in 1999–2010. The NHANES aims to evaluate health and nutritional status of representative populations in the United States. It collects information of approximately 5000 individuals regularly that carried out from 15 areas and examines in every two-year period since 1999, and utilizes a complex, multistage stratified probability sample based on selected counties, blocks, households, and persons within households. Well-trained professionals conduct interviews in person at their homes, and extensive physical examinations were conducted at mobile exam centers (MECs). This study was based on NHANES data, which received ethical approval from the National Center for Health Statistics (NCHS) Ethics Review Board. All participants gave written informed consent, and the survey followed the principles of the Declaration of Helsinki. The authors did not have access to any information that could identify individual participants at any point during or following data collection. The core dataset supporting the results reported in this manuscript is publicly available at Figshare via the following DOI: https://doi.org/10.6084/m9.figshare.31440001.

**Fig 1:**
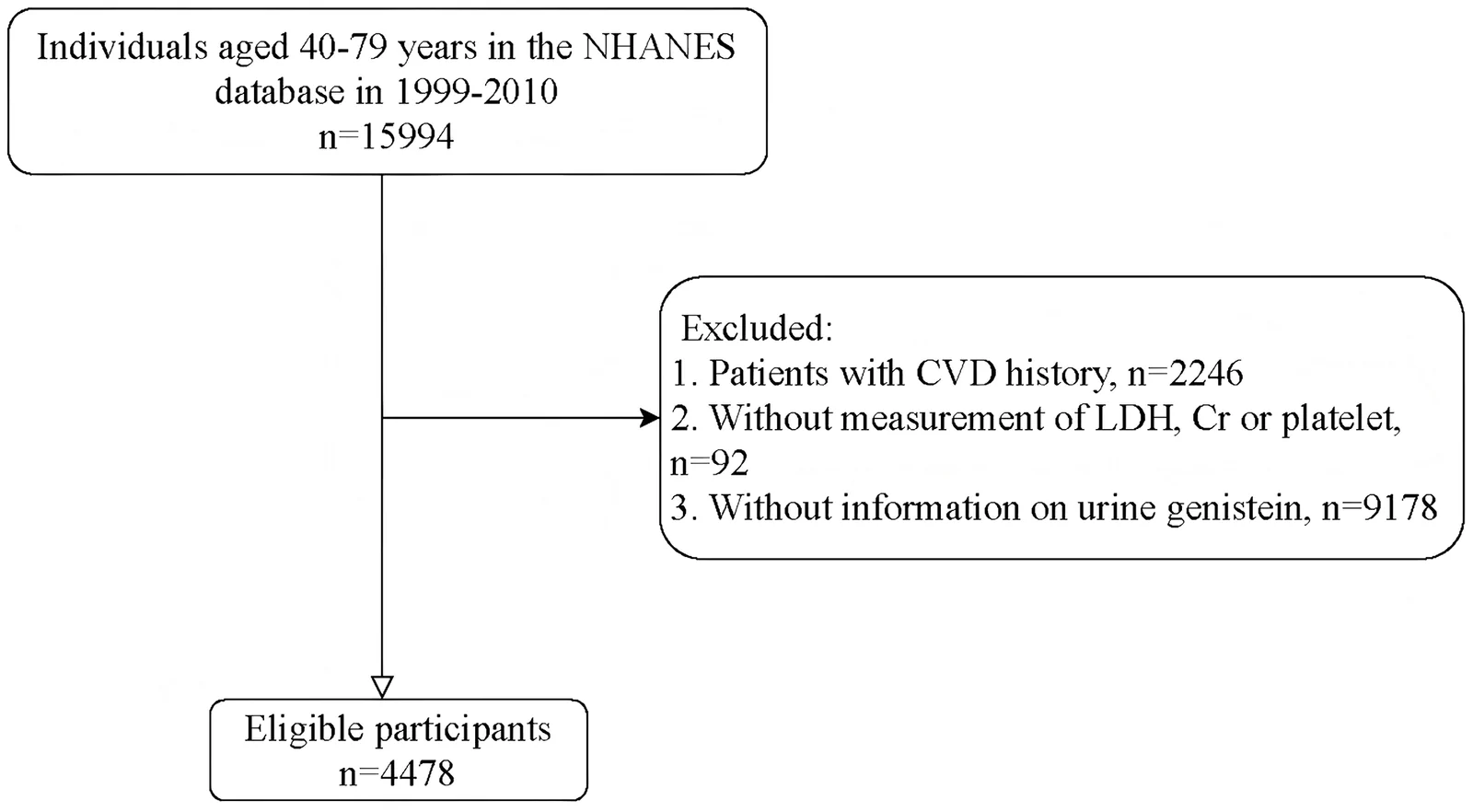
Flowchart of participants screening.

Individuals aged 40–79 years old in the database were included (n = 15994). The exclusion criteria were individuals with a history of cardiovascular disease (CVD) (n = 2246), individuals without information on lactate dehydrogenase (LDH), creatinine (Cr), or platelet count (n = 92), and individuals without a measurement of urine genistein (n = 9178). Finally, 4478 participants were eligible for further analysis (Fig 1).

### 2.2 Outcome indicators

ASCVD was defined according to the 2013 Guidelines for Cholesterol-Lowering Therapy to Reduce Atherosclerotic Cardiovascular Risk in Adults, published by the American College of Cardiology (ACC) and the American Heart Association (AHA) [17]. ASCVD is defined as the presence of at least one of the following conditions: coronary heart disease (CHD), angina pectoris, myocardial infarction, or stroke. The estimated 10-year ASCVD risk was calculated using a pooled cohort equation from the 2013 ACC/AHA guidelines [18]. This equation predicts the risk of stroke, nonfatal myocardial infarction, or death from coronary heart disease over the next 10 years in individuals aged 40–79 years with low-density lipoprotein cholesterol (LDL-C) levels <190 mg/dL and no history of ASCVD. The equation incorporates the following variables: age, sex, race, high-density lipoprotein cholesterol (HDL-C), total cholesterol, systolic blood pressure (SBP), use of antihypertensive medication, smoking status, and diabetes mellitus (DM). The estimated 10-year ASCVD risk classification was categorized as <7.5% and ≥7.5%, based on previous studies [19–20].

### 2.3 Laboratory

According to the laboratory guidelines of the NHANES, blood samples from the study subjects were collected by the staff of mobile examination centers (MECs) [21]. The blood samples were stored at approximately −30°C and transported to the Nutritional Biomarkers Unit of the Laboratory Sciences Division of NHANES for further analysis.

EASIX score was calculated by the formula-LDH (U/L)  ×  Cr (mg/dL) / platelet count (10^9^/L) and evaluated based on log2 transformed values.

Urinary genistein concentrations (μg/g) were normalized using urinary creatinine (urinary genistein concentration in ng/mL divided by urinary creatinine concentration in mg/dL). In epidemiologic studies, the 75th percentile is typically used as the criterion for the high-exposure group. Based on previous epidemiological studies that categorized urinary genistein concentrations using quartiles, we prespecified the upper quartile as the threshold for defining higher urinary genistein exposure [22–23]. The corresponding cutoff of 54.105 μg/g was determined as the 75th percentile of urinary genistein in the current analytic sample.

### 2.4 Covariates

Variables that could be potential confounding factors were also extracted from the database, including age, gender, race, educational level, marital status, poverty-to-income ratio (PIR), smoking, drinking, physical activity, total energy intake, diabetes mellitus (DM), hypertension, dyslipidemia, body mass index (BMI), antihypertensive medication use, lipid-lowering medication use, C-reactive protein (CRP) and uric acid (UA).

In the NHANES, dietary information of participants was collected through two 24-hour dietary recalls, and consists of daily diet and supplements [24]. Well trained interviewers conducted the first 24-hour recall interview in person in the MEC, and the second interview was performed through telephone or mail 3–10 days later. Dietary variables were calculated as the mean of two 24-hour dietary recalls when both recalls were available and were based on the available single recall when only one recall was available. During the NHANES household interviews, participants who claimed to have smoked fewer than 100 cigarettes in their lives were divided into non-smoking group. The pattern of alcohol consumption was also captured by questionnaires [25]. Overweight was defined according to WHO criteria as a BMI of ≥25 kg/m^2^. Physical activity was converted into metabolic equivalent (MET), which was calculated according to the information collected by physical activity questionnaire in the NHANES. Energy expenditure (MET·min) = recommended MET × exercise time of corresponding activity (min), with the cutoff value of 450 MET·min/week. Hypertension diagnosis was through self-reported hypertension or systolic blood pressure (SBP) ≥130 mmHg or diastolic blood pressure (DBP) ≥80 mmHg or using antihypertensive agent. Patients with total cholesterol ≥200 mg/dL (5.2 mmol/L) or triglycerides ≥150 mg/dL (1.7 mmol/L) or low-density lipoprotein cholesterol ≥130 mg/dL (3.4 mmol/L) or high-density lipoprotein cholesterol ≤40 mg/dL (1.0 mmol/L) or self-reported hypercholesteremia or receiving lipid-lowering therapy were identified as dyslipidemia [26]. DM was defined according to a self-reported diagnosis, the use of oral hypoglycemic agents or insulin, HbAlc ≥ 6.5%, a plasma glucose level ≥200 mg/dL at 2 hours after the oral glucose tolerance test, or a fasting glucose level ≥126 mg/dL.

### 2.5 Statistical analyses

Continuous variables were summarized as weighted means with standard errors (SEs) and were compared between the non-elevated and elevated estimated 10-year ASCVD risk classification groups using survey-weighted t tests. Categorical variables were summarized as unweighted numbers with weighted percentages and were compared using survey-weighted chi-square tests. In accordance with NHANES guidelines, appropriate MEC sample weights (WTMEC2YR and WTMEC4YR) were applied because multiple survey cycles were combined.

Covariates associated with 10-year ASCVD risk were identified using weighted logistic regression and included in multivariable models when *P* < 0.05. Weighted univariate and multivariable logistic regression analyses were performed to examine the association between EASIX and estimated 10-year ASCVD risk classification, reported as odds ratios (ORs) with 95% confidence intervals (CIs). Interaction between genistein and EASIX was assessed using a multiplicative interaction term, followed by stratified analyses. Additionally, restricted cubic splines (RCS) were utilized to evaluate the potential non-linear dose-response relationships. Subgroup analyses were conducted by age, BMI, diabetes, hypertension, and dyslipidemia.

Missing data for covariates with less than 20% missingness, including educational level, marital status, PIR, drinking status, physical activity, total energy intake, BMI, and CRP, were imputed using a random forest method prior to applying NHANES survey weights. Variables with substantial missingness were categorized as “unknown” when appropriate. Missing values were imputed only for covariates, whereas the main exposure variable, EASIX, and the primary outcome, elevated estimated 10-year ASCVD risk classification, were not imputed. All subsequent analyses incorporated NHANES survey weights. The proportions of missing values for key variables are provided in S1 Table, and the sensitivity analysis of participants’ characteristics before and after imputation of variables with missing values is provided in S2 Table. Statistical analyses were performed using R version 4.2.3.

## 3. Results

### 3.1 Characteristics of participants

A comparison of the characteristics of participants between the low 10-year ASCVD risk group (n = 2157) and the high 10-year ASCVD risk group (n = 2321) is presented in Table 1. The average age of total population was 54.26 years, 2185 (47.19%) of them were male. The mean values of EASIX in ASCVD <7.5% group were significantly lower than those in ASCVD ≥7.5% group (0.43 vs. 0.55). The average mean urine genistein concentrations in ASCVD <7.5% group were significantly higher than those in ASCVD ≥7.5% group (184.33 μg/g vs. 138.87 μg/g). In addition, the high-risk group tended to be older, male, hypertensive, diabetic, high cholesterol, and smokers (all *P* < 0.05).

**Table 1:** Characteristics of participants in low 10-year ASCVD risk group and high 10-year ASCVD risk group.

Variables	Total (n = 4478)	ASCVD <7.5% (n = 2157)	ASCVD ≥7.5% (n = 2321)	Statistics	*P*-value
Age, years, Mean (SE)	56.56 ± 11.06	48.86 ± 6.82	63.71 ± 9.29	t = −61.24	<0.001
Age, years, n (%)				χ^2^=1077.84	<0.001
<60	2604 (70.19)	1933 (92.85)	671 (40.07)		
≥60	1874 (29.81)	224 (7.15)	1650 (59.93)		
Gender, n (%)				χ^2^=327.83	<0.001
Male	2185 (47.19)	756 (35.60)	1429 (62.59)		
Female	2293 (52.81)	1401 (64.40)	892 (37.41)		
Race, n (%)				χ^2^=6.35	0.042
Non-Hispanic White	2186 (75.08)	1007 (73.75)	1179 (76.84)		
Non-Hispanic Black	884 (9.67)	438 (10.30)	446 (8.83)		
Others	1408 (15.25)	712 (15.95)	696 (14.33)		
Educational level, n (%)				χ^2^=83.44	<0.001
Below high school	1356 (17.40)	527 (13.30)	829 (22.85)		
High school	1059 (26.22)	485 (24.33)	574 (28.74)		
College and above	2063 (56.38)	1145 (62.37)	918 (48.41)		
Marital status, n (%)				χ^2^=23.52	<0.001
Married	2801 (67.46)	1370 (67.89)	1431 (66.89)		
Never married	315 (6.16)	194 (7.76)	121 (4.03)		
Others	1362 (26.38)	593 (24.35)	769 (29.08)		
PIR, n (%)				χ^2^=101.08	<0.001
≤1.3	1144 (15.08)	482 (12.80)	662 (18.11)		
1.3–3.5	1654 (32.96)	713 (28.11)	941 (39.40)		
>3.5	1680 (51.97)	962 (59.09)	718 (42.49)		
Smoking, n (%)				χ^2^=203.91	<0.001
No	2237 (50.32)	1397 (63.75)	840 (32.47)		
Yes	2241 (49.68)	760 (36.25)	1481 (67.53)		
Drinking, n (%)				χ^2^=0.01	0.908
No	1338 (26.18)	672 (26.25)	666 (26.08)		
Yes	3140 (73.82)	1485 (73.75)	1655 (73.92)		
Physical activity, met*minutes/week, n (%)				χ^2^=39.91	<0.001
<450	650 (15.90)	334 (17.14)	316 (14.24)		
≥450	2103 (52.08)	1100 (55.12)	1003 (48.03)		
Unknown	1725 (32.02)	723 (27.74)	1002 (37.72)		
Total energy intake, kcal, Mean (SE)	1996.51 ± 883.30	2051.72 ± 875.97	1944.22 ± 887.05	t = 4.06	<0.001
DM, n (%)				χ^2^=328.09	<0.001
No	3649 (87.14)	2009 (95.21)	1640 (76.42)		
Yes	829 (12.86)	148 (4.79)	681 (23.58)		
Hypertension, n (%)				χ^2^=442.56	<0.001
No	2150 (53.78)	1446 (68.72)	704 (33.93)		
Yes	2328 (46.22)	711 (31.28)	1617 (66.07)		
Dyslipidemia, n (%)				χ^2^=122.02	<0.001
No	930 (20.77)	620 (28.03)	310 (11.12)		
Yes	3548 (79.23)	1537 (71.97)	2011 (88.88)		
BMI, kg/m^2^, Mean (SE)	29.13 ± 6.32	28.78 ± 6.70	29.45 ± 5.93	t = −3.49	<.001
BMI overweight, kg/m^2^, n (%)				χ^2^=62.07	<0.001
<25	1146 (28.94)	647 (34.33)	499 (21.77)		
≥25	3332 (71.06)	1510 (65.67)	1822 (78.23)		
Hypotensive drugs, n (%)				χ^2^=292.72	<0.001
No	2843 (68.33)	1726 (80.76)	1117 (51.81)		
Yes	1635 (31.67)	431 (19.24)	1204 (48.19)		
Hypolipidemic drugs, n (%)				χ^2^=128.08	<0.001
No	3417 (78.04)	1859 (85.98)	1558 (67.50)		
Yes	1061 (21.96)	298 (14.02)	763 (32.50)		
CRP, mg/dL, Mean (SE)	0.47 ± 0.95	0.42 ± 0.77	0.51 ± 1.08	t = −3.09	0.002
UA, mg/dL, Mean (SE)	5.46 ± 1.41	5.11 ± 1.33	5.79 ± 1.40	t = −16.82	<.001
EASIX, Mean (SE)	0.49 ± 0.28	0.43 ± 0.22	0.55 ± 0.31	t = −14.91	<.001
Genistein, μg/g, Mean (SE)	164.81 ± 12.67	184.33 ± 18.38	138.87 ± 14.55	t = 2.02	0.046
Genistein, n (%)				χ^2^=1.01	0.315
≥54.105	1422 (33.32)	691 (34.08)	731 (32.30)		
<54.105	3056 (66.68)	1466 (65.92)	1590 (67.70)		

t: t test, χ^2^: chi-square test. ASCVD: atherosclerotic cardiovascular disease, SE: standard error, PIR: poverty-to-income ratio, DM: diabetes mellitus, BMI: body mass index, CRP: C-reactive protein, UA: uric acid, CRP: C-Reactive Protein.

### 3.2 Associations of genistein and EASIX with estimated 10-year ASCVD risk classification

Before we explored associations of genistein and EASIX with estimated 10-year ASCVD risk classification, covariates associated with 10-year ASCVD risk were screened (S3 Table). It was shown that age, educational level, marital status, PIR, physical activity, BMI, hypolipidemic drugs, CRP and UA were significantly associated with 10-year ASCVD risk (all *P* < 0.05).

According to the Table 2 after adjusting for all selected covariates (model 3), continuous EASIX maintained a robust and independent positive association with the odds of 10-year ASCVD risk. Specifically, for every 1-point increase in the EASIX score, the odds ratio for 10-year ASCVD risk increases by 40.7% (OR = 1.407; 95% CI: 1.208–1.640; *P* < 0.001). Regarding urinary genistein levels, no statistically significant independent association with estimated 10-year ASCVD risk classification was noted in the fully adjusted model, with an OR of 1.22 (95% CI: 0.97–1.55; *P* = 0.091) for those with genistein < 54.105 μg/g compared to the reference group (≥54.105). Sensitivity analyses based on EASIX tertiles and median dichotomization showed generally consistent associations between higher EASIX levels and elevated estimated 10-year ASCVD risk classification (S4 Table).

**Table 2:** Associations of genistein and EASIX with estimated 10-year ASCVD risk classification.

Variables	Model 1		Model 2		Model 3	
	OR (95% CI)	*P*-value	OR (95% CI)	*P*-value	OR (95% CI)	*P*-value
**EASIX**	1.982 (1.723–2.280)	<0.001	1.730 (1.489–2.010)	<0.001	1.407 (1.208–1.640)	<0.001
**Genistein**						
≥54.105	Ref		Ref		Ref	
<54.105	1.08 (0.92–1.27)	0.318	1.33 (1.05–1.68)	0.019	1.22 (0.97–1.55)	0.091

EASIX: the Endothelial Activation and Stress Index, ASCVD: atherosclerotic cardiovascular disease, OR: odds ratio, CI: confidence interval, Ref: reference. Model 1: unadjusted model; Model 2: adjusted for age, educational level, marital status and PIR; Model 3: adjusted for age, educational level, marital status, PIR, physical activity, hypolipidemic drugs, BMI, CRP and UA.

### 3.3 Potential interaction effect of genistein on association between EASIX and estimated 10-year ASCVD risk classification

**Fig 2:**
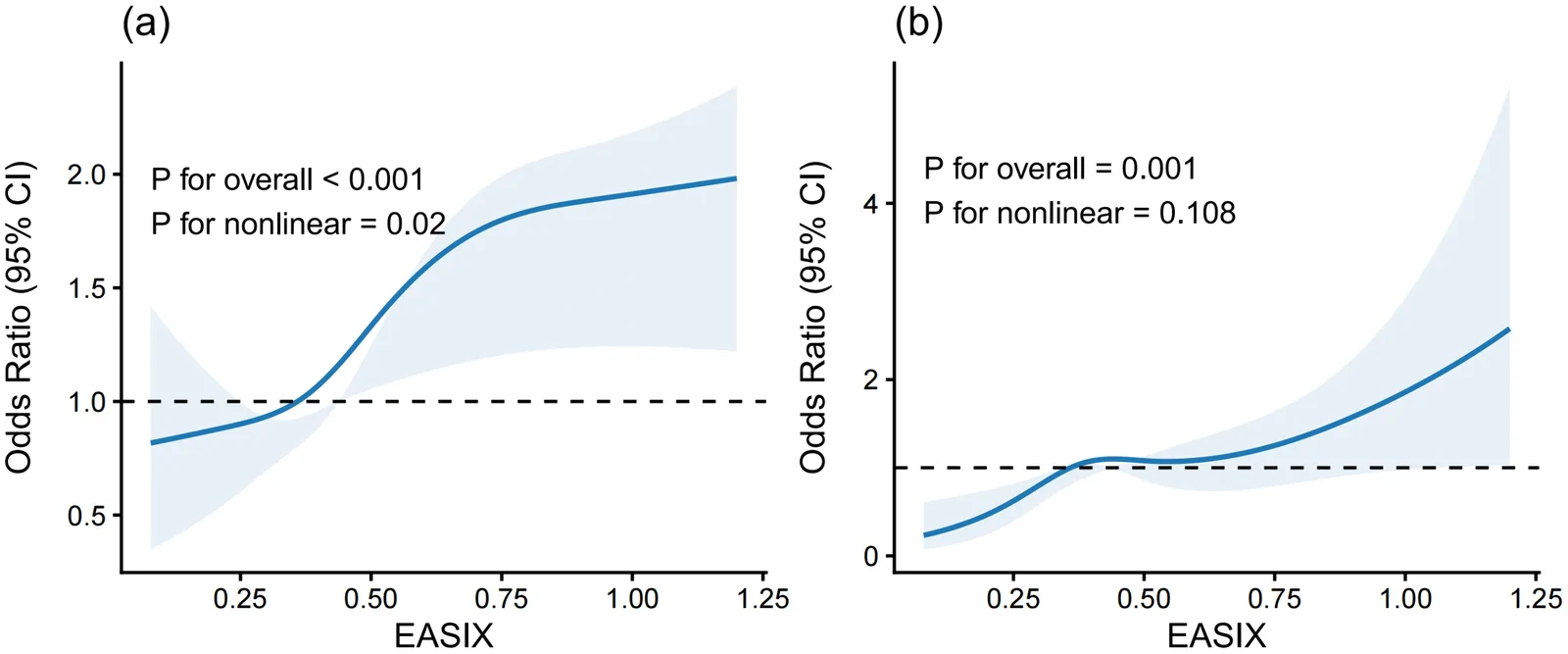
Dose-response associations of EASIX with estimated 10-year ASCVD risk classification stratified by urinary genistein levels: a restricted cubic spline analysis. Dose-response associations of continuous EASIX with the risk of ASCVD stratified by urinary genistein levels (a: Genistein < 54.105 ug/g; b: Genistein ≥ 54.105 ug/g). Data were fitted using restricted cubic splines in logistic regression models with 4 knots. The solid blue lines represent the multi-variable adjusted Odds Ratios (ORs), and the light blue shaded areas represent the corresponding 95% confidence intervals. The horizontal dashed line indicates the reference line where OR = 1.0. Models were fully adjusted for age, educational level, marital status, PIR, physical activity, hypolipidemic drugs, BMI, CRP and UA.; EASIX, Endothelial Activation Sequential Organ Failure Assessment; PIR, poverty-to-income ratio; CRP, C-reactive protein; UA: uric acid.

The multiplicative interaction effect between EASIX and genistein was significant after adjusting for covariates (OR = 1.66, 95% CI: 1.17–2.35, *P* = 0.005) (Table 3). Stratified analyses by urinary genistein levels revealed that continuous EASIX maintained a persistent positive association with estimated 10-year ASCVD risk classification in both strata, with a slight attenuation in risk magnitude observed in the higher genistein group (Table 4 and Fig 2). In the fully adjusted Model 3 in Table 4, each 1-unit increase in EASIX was associated with higher odds of being classified as having elevated estimated 10-year ASCVD risk classification among participants with lower creatinine-corrected urinary genistein levels (< 54.105 μg/g) (OR = 1.397; 95% CI: 1.202–1.624; *P* < 0.001). In comparison, this adverse effect was slightly blunted but remained statistically significant among those with higher genistein levels (≥ 54.105 μg/g), where each 1-unit increase in EASIX was associated with a 36.0% increase in the odds of estimated 10-year ASCVD (OR = 1.360; 95% CI: 1.066–1.736; *P* = 0.015). However, despite the statistically significant multiplicative interaction, the difference between the stratum-specific estimates was modest (OR = 1.397 vs. 1.360). Sensitivity analyses using tertile-based and median-based categorizations of EASIX showed results that were broadly consistent with those of the continuous EASIX analysis, although the magnitude and statistical significance of the associations varied across urinary genistein strata (S5 Table).

**Table 3:** Potential interaction effect of genistein on association between EASIX and estimated 10-year ASCVD risk classification.

Variables	Model 1		Model 2		Model 3	
	OR (95% CI)	*P*-value	OR (95% CI)	*P*-value	OR (95% CI)	*P*-value
**EASIX**	1.89 (1.56–2.29)	<0.001	1.30 (0.99–1.72)	0.062	1.04 (0.77–1.40)	0.793
**Genistein**	0.94 (0.75–1.16)	0.553	0.98 (0.73–1.32)	0.909	0.94 (0.71–1.25)	0.669
EASIX *Genistein	1.30 (1.03–1.65)	0.028	1.75 (1.24–2.46)	0.002	1.66 (1.17–2.35)	0.005

EASIX: the Endothelial Activation and Stress Index, ASCVD: atherosclerotic cardiovascular disease, OR: odds ratio, CI: confidence interval. Model 1: unadjusted model; Model 2: adjusted for age, educational level, marital status and PIR; Model 3: adjusted for age, educational level, marital status, PIR, physical activity, hypolipidemic drugs, BMI, CRP and UA.

**Table 4:** Associations of EASIX with estimated 10-year ASCVD risk classification stratified by urinary genistein levels.

Variables	Model 1		Model 2		Model 3	
	OR (95% CI)	*P*-value	OR (95% CI)	*P*-value	OR (95% CI)	*P*-value
Genistein ≥54.105 (n = 1422)						
EASIX	1.729 (1.393–2.147)	<0.001	1.370 (1.078–1.741)	0.012	1.360 (1.066–1.736)	0.015
Genistein <54.105 (n = 3056)						
EASIX	1.730 (1.489–2.010)	<0.001	1.407 (1.208–1.640)	<0.001	1.397 (1.202–1.624)	<0.001

EASIX: the Endothelial Activation and Stress Index, ASCVD: atherosclerotic cardiovascular disease, OR: odds ratio, CI: confidence interval. Model 1: unadjusted model; Model 2: adjusted for age, educational level, marital status and PIR; Model 3: adjusted for age, educational level, marital status, PIR, physical activity, hypolipidemic drugs, BMI, CRP and UA.

Consistently, restricted cubic spline (RCS) curves further illustrated these patterns (Fig 2). In the lower genistein group, EASIX displayed a significant non-linear S-shaped relationship (*P*_overall_ < 0.001, *P*_nonlinear_ = 0.020), whereas a predominantly linear risk escalation was observed in the higher genistein group (*P*_overall_ = 0.001, *P*_nonlinear_ = 0.108).

### 3.4 Potential interaction effect of genistein on association between EASIX and estimated 10-year ASCVD risk classification in different subgroups

**Fig 3:**
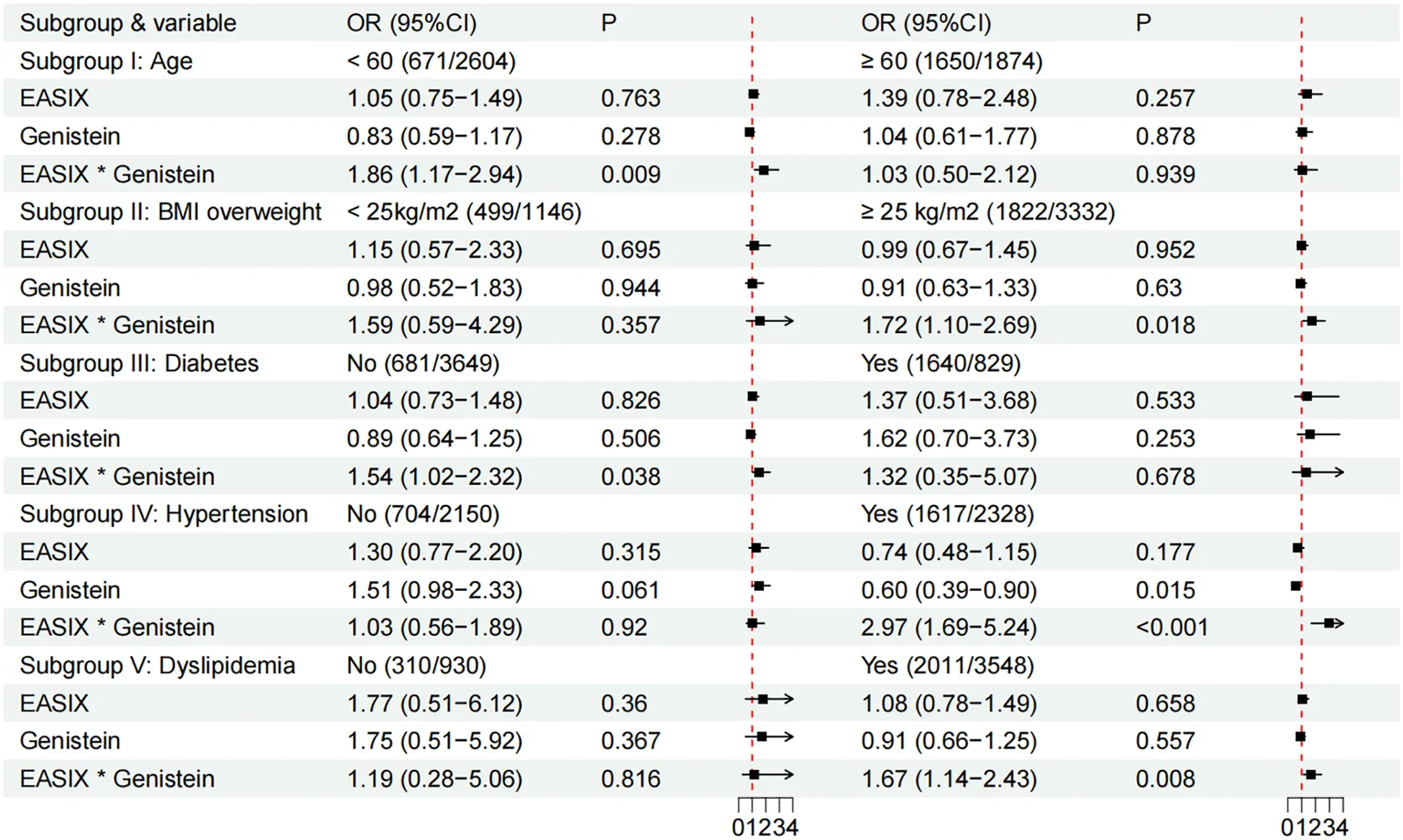
Association between EASIX and estimated 10-year ASCVD risk classification under different genistein levels in different subgroups. OR: odds ratio; CI: confidence interval; Ref: Reference; Age < 60: adjusted Education, Marriage, PIR, Physical activity, Drug for dyslipidemia, BMI, CRP, Uric acid; BMI ≥ 25 kg/m2: adjusted Age, Education, Marriage, PIR, Physical activity, Drug for dyslipidemia, CRP, Uric acid; Diabetes = No: adjusted Age, Education, Marriage, PIR, Physical activity, Drug for dyslipidemia, BMI, CRP, Uric acid; Hypertension = Yes: adjusted Age, Education, Marriage, PIR, Physical activity, Drug for dyslipidemia, BMI, CRP, Uric acid; Dyslipidemia = Yes: adjusted Age, Education, Marriage, PIR, Physical activity, Drug for dyslipidemia, BMI, CRP, Uric acid.

Moreover, we explored the potential interaction between genistein and the association of EASIX with estimated 10-year ASCVD risk classification (Table 5). Significant multiplicative interactions were observed in several subgroups, including adults aged < 60 years (OR = 1.86, 95%CI: 1.17–2.94, *P* = 0.009), those with BMI ≥ 25 kg/m² (OR = 1.72, 95%CI: 1.10–2.69, *P* = 0.018), without diabetes (OR = 1.54, 95%CI: 1.02–2.32, *P* = 0.038), and with hypertension (OR = 2.97, 95%CI: 1.69–5.24, *P* < 0.001) or dyslipidemia (OR = 1.67, 95%CI: 1.14–2.43, *P* = 0.008). When stratified by genistein level, the positive association between EASIX and estimated ASCVD risk classification was more apparent among participants with lower genistein levels, whereas the association was attenuated in those with higher levels (Fig 3).

**Table 5:** Potential interaction effect of genistein on the association between EASIX and estimated 10-year ASCVD risk classification across different subgroups.

Variables	Model 3			
	OR (95% CI)	*P*-value	OR (95% CI)	*P*-value
	Age < 60 (n = 2604)		Age ≥ 60 (n = 1874)	
**EASIX**	1.05 (0.75–1.49)	0.763	1.39 (0.78–2.48)	0.257
**Genistein**	0.83 (0.59–1.17)	0.278	1.04 (0.61–1.77)	0.878
EASIX * Genistein	1.86 (1.17–2.94)	0.009	1.03 (0.50–2.12)	0.939
	BMI < 25 kg/m^2^ (n = 1146)		BMI ≥ 25 kg/m^2^ (n = 3332)	
**EASIX**	1.15 (0.57–2.33)	0.695	0.99 (0.67–1.45)	0.952
**Genistein**	0.98 (0.52–1.83)	0.944	0.91 (0.63–1.33)	0.630
EASIX * Genistein	1.59 (0.59–4.29)	0.357	1.72 (1.10–2.69)	0.018
	Non-DM (n = 3649)		DM (n = 829)	
**EASIX**	1.04 (0.73–1.48)	0.826	1.37 (0.51–3.68)	0.533
**Genistein**	0.89 (0.64–1.25)	0.506	1.62 (0.70–3.73)	0.253
EASIX * Genistein	1.54 (1.02–2.32)	0.038	1.32 (0.35–5.07)	0.678
	Non-hypertension (n = 2150)		Hypertension (n = 2328)	
**EASIX**	1.30 (0.77–2.20)	0.315	0.74 (0.48–1.15)	0.177
**Genistein**	1.51 (0.98–2.33)	0.061	0.60 (0.39–0.90)	0.015
EASIX * Genistein	1.03 (0.56–1.89)	0.920	2.97 (1.69–5.24)	<0.001
	Non-dyslipidemia (n = 930)		Dyslipidemia (n = 3548)	
**EASIX**	1.77 (0.51–6.12)	0.360	1.08 (0.78–1.49)	0.658
**Genistein**	1.75 (0.51–5.92)	0.367	0.91 (0.66–1.25)	0.557
EASIX * Genistein	1.19 (0.28–5.06)	0.816	1.67 (1.14–2.43)	0.008

EASIX: the Endothelial Activation and Stress Index, ASCVD: atherosclerotic cardiovascular disease, OR: odds ratio, CI: confidence interval. Model 3: adjusted for age, educational level, marital status, PIR, physical activity, hypolipidemic drugs, BMI, CRP and UA.

## 4. Discussion

In this cross-sectional study of NHANES participants aged 40–79 years, we examined the associations of urinary genistein and EASIX with estimated 10-year ASCVD risk classification and assessed whether the association between EASIX and estimated ASCVD risk classification varied across urinary genistein strata. Higher EASIX levels were significantly associated with greater odds of elevated estimated 10-year ASCVD risk classification. Although a statistically significant multiplicative interaction between EASIX and urinary genistein was observed, the stratum-specific estimates were closely comparable across the lower and higher urinary genistein strata. This suggests that the magnitude of the between-stratum difference was relatively small and that its clinical relevance remains uncertain. Therefore, the interaction finding should be interpreted cautiously as exploratory and hypothesis-generating and requires confirmation in independent cohorts.

Endothelial dysfunction is the first clinical correlate of atherosclerosis identified, and since most cardiovascular diseases are related to or a direct consequence of atherosclerosis, endothelial dysfunction is an early predictor of subsequent cardiovascular events or mortality [27]. The EASIX has emerged as a promising biomarker for endothelial injury in recent years [28]. Sang et al. [29] based on the Medical Information Mart for Intensive Care-IV (MIMIC-IV) database and suggested that an elevated EASIX score is significantly correlated with increased 30-day mortality risk in patients with acute myocardial infarction. Finke et al. [30] validated that EASIX is a potential biomarker to predict mortality of coronary artery disease patients. Another MIMIC study showed that a high EASIX was significantly associated with an increased risk of 28-day and 90-day all-cause mortality in patients with sepsis [31]. Compared with previous studies mainly conducted in clinical populations, our study extends the evidence by showing an association between EASIX and elevated estimated 10-year ASCVD risk classification. However, because the outcome was based on an estimated risk score rather than observed cardiovascular events, these findings should be interpreted as associations with estimated ASCVD risk classification rather than evidence of incident ASCVD risk.

Both epidemiological studies and animal and in vitro studies have suggested that genistein may have a protective role in cardiovascular disease [15]. A study conducted by Huang et al. [32] indicated that the hypothesis that genistein protects vascular endothelial cells against the pro-atherosclerotic stressor, oxidized LDL, by inducing antioxidant enzymes and preventing apoptosis. Also, Wei et al. [33] suggested that genistein blocked Δ9-tetrahydrocannabinol-induced endothelial dysfunction in wire myograph, reduced atherosclerotic plaque, and had minimal penetration of the central nervous system in mice. Currently, accumulating evidence has revealed that endothelial cell apoptosis is the first step of atherosclerosis. Study preseted natural medicines has the potential in the treatment of atherosclerosis by inhibiting endothelial cell apoptosis [34].

Based on this evidence, we hypothesized that the association between EASIX and elevated estimated 10-year ASCVD risk classification may differ across urinary genistein strata. By evaluating the multiplicative interaction between urinary genistein and EASIX, we observed that the association between higher EASIX and elevated estimated 10-year ASCVD risk classification was more evident among participants with urinary genistein levels <54.105 μg/g, whereas it appeared less pronounced among those with urinary genistein levels ≥54.105 μg/g. This finding suggests a difference in the association between EASIX and estimated ASCVD risk classification across urinary genistein strata, rather than evidence of a protective or causal effect of genistein.

The underlying biological mechanisms for the observed difference across urinary genistein strata may be complex. EASIX is calculated using LDH, creatinine, and platelet count, and these components may reflect endothelial stress, tissue injury, renal dysfunction, and systemic inflammation. Inflammation is an important risk factor that induces endothelial dysfunction and further mediates the initiation and plaque progression of atherosclerosis (AS) [35]. For example, LDH and its major metabolite lactate have been reported to drive nasopharyngeal carcinoma progression by regulating TGF-β-activated kinase 1 and its downstream NF-κB signaling [36]. Lower platelet counts, another component of EASIX, can be indicative of increased platelet consumption or destruction, which is often seen in severe systemic inflammation or disseminated intravascular coagulation [37].

Genistein belongs to the flavonoid family, within the subgroup of isoflavones, and is a phytoestrogen mainly derived from legumes. It is a naturally occurring compound with a chemical structure similar to mammalian estrogens. The anti-inflammatory activity of genistein has been reported in both in vitro and in vivo studies, with potential mechanisms including inhibition of nuclear factor kappa-B (NF-κB), prostaglandins (PGs), pro-inflammatory cytokines, inducible nitric oxide synthase (iNOS), reactive oxygen species (ROS), and free radical scavenging activity [38]. In addition, genistein has been reported to protect vascular endothelial cells against oxidized LDL-induced injury by inducing antioxidant enzymes and preventing apoptosis [32]. The NF-κB signaling pathway can regulate the inflammatory and redox states of vascular endothelial cells and may partly mediate vascular endothelial dysfunction [39].

Taken together, inflammation and oxidative stress may provide a plausible biological context for the observed difference in the association between EASIX and ASCVD risk across urinary genistein strata. Because EASIX-related pathways may reflect endothelial stress and systemic inflammation, while genistein-related pathways may involve anti-inflammatory and antioxidant activity, these processes may partly overlap in the context of ASCVD risk. However, this interpretation remains speculative and should be regarded as hypothesis-generating.

This study is the first to examine the association between EASIX and estimated ASCVD risk classification, as well as the interaction between genistein and EASIX in relation to this risk, in a general population in the United States, which may provide useful information for ASCVD risk monitoring and prevention. The study population was derived from the NHANES database, which uses a large, nationally representative sample obtained through multistage complex sampling. However, several limitations should be acknowledged. First, the cross-sectional design of this study precludes the establishment of temporal relationships or causal inferences. Reverse causation cannot be excluded, as individuals with higher estimated cardiovascular risk may have modified their dietary habits, including soy food consumption, which could in turn influence urinary genistein levels. Second, urinary genistein was measured at a single time point and may primarily reflect recent dietary exposure rather than long-term intake. Third, the primary outcome was an estimated 10-year ASCVD risk classification rather than observed cardiovascular events. Because the pooled cohort equation incorporates traditional cardiovascular risk factors, the use of this derived outcome may introduce model redundancy, over-adjustment, and potential mathematical coupling. Given that the outcome is partially constructed from variables included in the adjustment set, the observed associations should be interpreted as associations with a composite risk score rather than as evidence of independent cardiovascular risk prediction. Fourth, although survey weights and adjustment for multiple covariates were applied, residual confounding cannot be completely excluded. Urinary genistein may reflect broader dietary and lifestyle patterns, including soy intake and overall diet quality, rather than an isolated exposure. Therefore, residual confounding from unmeasured or incompletely measured dietary and lifestyle factors may remain. Fifth, subgroup analyses were exploratory, and no correction for multiple testing was performed. Isolated significant interactions should therefore be interpreted cautiously, particularly because no consistent interaction pattern was observed across subgroups. Sixth, the exclusion of nearly 60% of otherwise eligible participants because of unavailable urinary genistein measurements may have introduced selection bias and limited the generalizability of the findings. Finally, the clinical utility and incremental predictive value of EASIX beyond established ASCVD risk factors were not assessed.

## 5. Conclusion

Higher EASIX was associated with a higher likelihood of elevated estimated 10-year ASCVD risk classification among United States adults. The association between EASIX and estimated ASCVD risk differed across urinary genistein strata, appearing weaker among participants with higher urinary genistein levels. These findings should be interpreted as hypothesis-generating, may inform future research on endothelial stress–related biomarkers, and require confirmation in prospective studies with observed ASCVD events.

## Supporting information

S1 Table Variables with missing values and missing proportion. PIR: poverty-to-income ratio, BMI: body mass index, CRP: C-reactive protein.
(DOCX)

S2 Table Sensitivity analysis of participants’ characteristics before and after the interpolation of variables with missing values. t: t test, χ2: chi-square test. PIR: poverty-to-income ratio, SE: standard error, BMI: body mass index, CRP: C-reactive protein.
(DOCX)

S3 Table Covariates associated with estimated 10-year ASCVD risk classification. ASCVD: atherosclerotic cardiovascular disease, OR: odds ratio, CI: confidence interval, Ref: reference, PIR: poverty-to-income ratio, BMI: body mass index, CRP: C-reactive protein, UA: uric acid.
(DOCX)

S4 Table Odds ratios for estimated 10-year ASCVD classification risk according to EASIX in continuous, tertile, and dichotomized forms. EASIX: the Endothelial Activation and Stress Index, ASCVD: atherosclerotic cardiovascular disease, OR: odds ratio, CI: confidence interval. Model 1: unadjusted model; Model 2: adjusted for age, educational level, marital status and PIR; Model 3: adjusted for age, educational level, marital status, PIR, physical activity, hypolipidemic drugs, BMI, CRP and UA.
(DOCX)

S5 Table Associations of EASIX (continuous, tertile, and dichotomized forms) with estimated 10-year ASCVD risk classification stratified by urinary genistein levels. EASIX: the Endothelial Activation and Stress Index, ASCVD: atherosclerotic cardiovascular disease, OR: odds ratio, CI: confidence interval. Model 1: unadjusted model; Model 2: adjusted for age, educational level, marital status and PIR; Model 3: adjusted for age, educational level, marital status, PIR, physical activity, hypolipidemic drugs, BMI, CRP and UA.
(DOCX)
