# Supplementary material for: Interaction between genistein and the endothelial activation and stress index in relation to estimated 10-year atherosclerotic cardiovascular disease risk classification
Source: PLoS One. 2026 Aug 19;21(8):e0356477. doi: 10.1371/journal.pone.0356477 (PMC13489419; doi:10.1371/journal.pone.0356477)
Supplement: Supplementary file 1 [file pone.0356477.s001.docx]

**S1 Table . Variables with missing values and missing proportion**

| Variables | N (%) |
| --- | --- |
| Educational level | 5 (0.11) |
| Marital status | 66 (1.47) |
| PIR | 355 (7.93) |
| Drinking | 202 (4.51) |
| Physical activity | 202 (4.51) |
| Total energy intake | 1725 (38.52) |
| BMI | 116 (2.59) |
| CRP | 45 (1) |

PIR: poverty-to-income ratio, BMI: body mass index, CRP: C-reactive protein.
