# Supplementary material for: Interaction between genistein and the endothelial activation and stress index in relation to estimated 10-year atherosclerotic cardiovascular disease risk classification
Source: PLoS One. 2026 Aug 19;21(8):e0356477. doi: 10.1371/journal.pone.0356477 (PMC13489419; doi:10.1371/journal.pone.0356477)
Supplement: Supplementary file 2 [file pone.0356477.s002.docx]

**S2 Table. Sensitivity analysis of participants’ characteristics before and after the interpolation of variables with missing values**

| Variables | After interpolation | Before interpolation | Statistics | *P* |
| --- | --- | --- | --- | --- |
| Educational level, n (%) |  |  | χ^2^=2.75 | 0.253 |
| Below high school | 1352 (17.38) | 1356 (17.40) |  |  |
| High school | 1059 (26.24) | 1059 (26.22) |  |  |
| College and above | 2062 (56.38) | 2063 (56.38) |  |  |
| Marital status, n (%) |  |  | χ^2^=3.57 | 0.168 |
| Married | 2758 (67.35) | 2801 (67.46) |  |  |
| Never married | 312 (6.25) | 315 (6.16) |  |  |
| Others | 1342 (26.40) | 1362 (26.38) |  |  |
| PIR, n (%) |  |  | χ^2^=0.45 | 0.800 |
| ≤1.3 | 1054 (15.14) | 1144 (15.08) |  |  |
| 1.3-3.5 | 1502 (32.81) | 1654 (32.96) |  |  |
| >3.5 | 1567 (52.05) | 1680 (51.97) |  |  |
| Drinking, n (%) |  |  | χ^2^=0.01 | 0.910 |
| No | 1290 (26.20) | 1338 (26.18) |  |  |
| Yes | 2986 (73.80) | 3140 (73.82) |  |  |
| Total energy intake, kcal, Mean (SE) | 2123.31 (17.85) | 2127.72 (17.34) | t=-1.44 | 0.154 |
| BMI, kg/m^2^, Mean (SE) | 28.80 (0.13) | 28.81 (0.13) | t=-1.01 | 0.314 |
| CRP, mg/dL, Mean (SE) | 0.42 (0.01) | 0.42 (0.01) | t=1.16 | 0.248 |

t: t test, χ2: chi-square test

PIR: poverty-to-income ratio, SE: standard error, BMI: body mass index, CRP: C-reactive protein.
