# Supplementary material for: Interaction between genistein and the endothelial activation and stress index in relation to estimated 10-year atherosclerotic cardiovascular disease risk classification
Source: PLoS One. 2026 Aug 19;21(8):e0356477. doi: 10.1371/journal.pone.0356477 (PMC13489419; doi:10.1371/journal.pone.0356477)
Supplement: Supplementary file 3 [file pone.0356477.s003.docx]

**S3 Table. Covariates associated with estimated 10-year ASCVD risk classification**

| Variables | OR (95% CI) | *P* |
| --- | --- | --- |
| Age | 1.20 (1.19-1.22) | **<0.001** |
| Educational level |  |  |
| Below high school | Ref |  |
| High school | 0.69 (0.56-0.85) | **<0.001** |
| College and above | 0.45 (0.37-0.55) | **<0.001** |
| Marital status |  |  |
| Married | Ref |  |
| Never married | 0.53 (0.39-0.70) | **<0.001** |
| Others | 1.21 (1.01-1.45) | **0.034** |
| PIR |  |  |
| ≤1.3 | Ref |  |
| 1.3-3.5 | 0.99 (0.82-1.19) | 0.914 |
| >3.5 | 0.51 (0.43-0.60) | **<0.001** |
| Drinking |  |  |
| No | Ref |  |
| Yes | 1.01 (0.87-1.17) | 0.908 |
| Physical activity |  |  |
| <450 | Ref |  |
| ≥450 | 1.05 (0.87-1.27) | 0.621 |
| Unknown | 1.64 (1.36-1.98) | **<0.001** |
| Total energy intake | 1.00 (1.00-1.00) | 0.156 |
| BMI | 1.03 (1.02-1.04) | **<0.001** |
| Hypolipidemic drugs |  |  |
| No | Ref |  |
| Yes | 2.95 (2.41-3.61) | **<0.001** |
| CRP | 1.13 (1.01-1.25) | **0.027** |
| UA | 1.48 (1.39-1.57) | **<0.001** |

The bold P values represented statistically significant.

ASCVD: atherosclerotic cardiovascular disease, OR: odds ratio, CI: confidence interval, Ref: reference, PIR: poverty-to-income ratio, BMI: body mass index, CRP: C-reactive protein, UA: uric acid
