# Supplementary material for: Interaction between genistein and the endothelial activation and stress index in relation to estimated 10-year atherosclerotic cardiovascular disease risk classification
Source: PLoS One. 2026 Aug 19;21(8):e0356477. doi: 10.1371/journal.pone.0356477 (PMC13489419; doi:10.1371/journal.pone.0356477)
Supplement: Supplementary file 4 [file pone.0356477.s004.docx]

**S4 Table. Odds ratios for estimated 10-year ASCVD risk classification according to EASIX in continuous, tertile, and dichotomized forms**

| EASIX |  | Crude model | | Model 1 | | Model 2 | | Model 3 | |
| --- | --- | --- | --- | --- | --- | --- | --- | --- | --- |
|  |  | **OR(95%CI)** | **P** | **OR(95%CI)** | **P** | **OR(95%CI)** | **P** | **OR(95%CI)** | **P** |
| EASIX (Continuous) | - | 1.982 (1.723-2.280) | <0.001 | 1.730 (1.489-2.010) | <0.001 | 1.407 (1.208-1.640) | <0.001 | 1.397 (1.202-1.624) | <0.001 |
| EASIX (Tertiles) | <0.364 | Reference | - | Reference | - | Reference | - | Reference | - |
|  | 0.364-0.523 | 1.549 (1.249-1.922) | <0.001 | 1.515 (1.121-2.048) | 0.008 | 1.298 (0.935-1.802) | 0.123 | 1.300 (0.935-1.807) | 0.123 |
|  | >0.523 | 3.095 (2.510-3.816) | <0.001 | 2.606 (2.004-3.389) | <0.001 | 1.932 (1.435-2.601) | <0.001 | 1.920 (1.424-2.588) | <0.001 |
| EASIX (Dichotomized) | ≤ 0.427 | Reference | - | Reference | - | Reference | - | Reference | - |
|  | > 0.427 | 2.464 (2.101-2.890) | <0.001 | 2.195 (1.761-2.735) | <0.001 | 1.744 (1.369-2.222) | <0.001 | 1.741 (1.366-2.219) | <0.001 |

EASIX: the Endothelial Activation and Stress Index, ASCVD: atherosclerotic cardiovascular disease, OR: odds ratio, CI: confidence interval. Model 1: unadjusted model; Model 2: adjusted for age, educational level, marital status and PIR; Model 3: adjusted for age, educational level, marital status, PIR, physical activity, hypolipidemic drugs, BMI, CRP and UA.
