# Supplementary material for: Interaction between genistein and the endothelial activation and stress index in relation to estimated 10-year atherosclerotic cardiovascular disease risk classification
Source: PLoS One. 2026 Aug 19;21(8):e0356477. doi: 10.1371/journal.pone.0356477 (PMC13489419; doi:10.1371/journal.pone.0356477)
Supplement: Supplementary file 5 [file pone.0356477.s005.docx]

**S5 Table. Associations of EASIX (continuous, tertile, and dichotomized forms) with estimated 10-year ASCVD risk classification stratified by urinary genistein levels**

|  |  | Crude model |  | Model 1 |  | Model 2 |  | Model 3 |  |
| --- | --- | --- | --- | --- | --- | --- | --- | --- | --- |
| Genistein ≥ 54.105ug/g (n=1422) |  |  |  |  |  |  |  |  |  |
|  |  | OR(95%CI) | P | OR(95%CI) | P | OR(95%CI) | P | OR(95%CI) | P |
| EASIX (Continuous) | - | 1.892 (1.625-2.203) | <0.001 | 1.729 (1.393-2.147) | <0.001 | 1.370 (1.078-1.741) | 0.012 | 1.360 (1.066-1.736) | 0.015 |
| EASIX (Tertiles) | <0.364 | Reference | - | Reference | - | Reference | - | Reference | - |
|  | 0.364-0.523 | 1.892 (1.362-2.628) | <0.001 | 1.586 (1.012-2.485) | 0.047 | 1.211 (0.754-1.945) | 0.431 | 1.242 (0.767-2.012) | 0.382 |
|  | >0.523 | 3.121 (2.373-4.104) | <0.001 | 2.458 (1.665-3.629) | <0.001 | 1.576 (1.047-2.373) | 0.032 | 1.584 (1.046-2.398) | 0.033 |
| EASIX (Dichotomized) | ≤ 0.427 | Reference | - | Reference | - | Reference | - | Reference | - |
|  | > 0.427 | 1.889 (1.560-2.287) | <0.001 | 1.305 (0.981-1.736) | 0.071 | 0.983 (0.713-1.355) | 0.916 | 0.973 (0.699-1.354) | 0.870 |
| Genistein < 54.105 ug/g (n=3056) |  |  |  |  |  |  |  |  |  |
| EASIX (Continuous) | - | 1.982 (1.723-2.280) | <0.001 | 1.730 (1.489-2.010) | <0.001 | 1.407 (1.208-1.640) | <0.001 | 1.397 (1.202-1.624) | <0.001 |
| EASIX (Tertiles) | <0.364 | Reference | - | Reference | - | Reference | - | Reference | - |
|  | 0.364-0.523 | 1.549 (1.249-1.922) | <0.001 | 1.515 (1.121-2.048) | 0.008 | 1.298 (0.935-1.802) | 0.123 | 1.300 (0.935-1.807) | 0.123 |
|  | >0.523 | 3.095 (2.510-3.816) | <0.001 | 2.606 (2.004-3.389) | <0.001 | 1.932 (1.435-2.601) | <0.001 | 1.920 (1.424-2.588) | <0.001 |
| EASIX (Dichotomized) | ≤ 0.427 | Reference | - | Reference | - | Reference | - | Reference | - |
|  | > 0.427 | 2.464 (2.101-2.890) | <0.001 | 2.195 (1.761-2.735) | <0.001 | 1.744 (1.369-2.222) | <0.001 | 1.741 (1.366-2.219) | <0.001 |

EASIX: the Endothelial Activation and Stress Index, ASCVD: atherosclerotic cardiovascular disease, OR: odds ratio, CI: confidence interval. Model 1: unadjusted model; Model 2: adjusted for age, educational level, marital status and PIR; Model 3: adjusted for age, educational level, marital status, PIR, physical activity, hypolipidemic drugs, BMI, CRP and UA.
